# Cranial nerve involvement in mucormycosis in post-COVID patients: a case series

**DOI:** 10.1186/s43055-022-00700-8

**Published:** 2022-01-24

**Authors:** Neeti Gupta, Saurabh Dembla

**Affiliations:** Department of Radiology, Sir J.J. Group of Hospitals, Mumbai, India

**Keywords:** Rhinosinocerebral mucormycosis, Novel COVID-19 disease, Cranial nerves, Neuritis, Fungal, Garcin syndrome

## Abstract

**Background:**

One of the largest outbreaks of rhinosinocerebral mucormycosis (RSCM) occurred in India close to the second wave of the SARS-CoV-2 infection. RSCM is a rare infection caused by several fungal species occurring in immunocompromised subjects. Mucor shows a high propensity to invade the central nervous system. There have been limited studies, mostly isolated case reports, on the neurological manifestations of RSCM. The outbreak of mucormycosis infection was thus the most opportune to study the neurological manifestations and cranial nerve involvement in mucormycosis in greater depths.

**Aim of the study:**

The purpose of the study was to investigate and review the involvement of cranial nerves in a series of cases of rhinosinocerebral mucormycosis associated with the novel coronavirus disease caused by SARS-CoV-2.

**Results:**

It was a retrospective cross-sectional study of seven patients who were undergoing treatment of RSCM with a recent history of coronavirus disease caused by SARS-CoV-2 infection within the last 3 months. Patients with cranial nerve involvement were identified by magnetic resonance imaging (MRI) at a single institution. Demographic details of the patients, clinical presentation, imaging, microbiological and pathological findings were recorded. All subjects had two or more cranial nerves affected by fungal infection. The most commonly involved cranial nerve was found to be the optic nerve followed by the trigeminal nerve and its branches. We document three cases with extensive involvement of the inferior alveolar branch of the mandibular division of the trigeminal nerve (V3), a previously unreported finding. In one case, in addition to the second and fifth cranial nerves, the third, fourth, sixth, seventh, eighth, and twelfth cranial nerves were involved without any sensory or motor long tract involvement, suggestive of Garcin syndrome secondary to intracranial abscesses and skull base osteomyelitis due to invasive fungal infection. This case is of rare occurrence in the literature, and our study provides one such example.

**Conclusion:**

Cranial nerve involvement in patients of mucormycosis tends to have a poor prognosis, both cosmetic and functional. Radical surgeries and aggressive medical management is needed in such cases to improve the outcome.

## Background

Since the first reported case of coronavirus infection in December 2019, the disease has spread to almost all countries of the world.

Coronavirus disease produces protean manifestations and complications. Several uncommon phenomena have been reported in association with the disease. These phenomena include several types of co-infections [1], new-onset diabetes, strokes in the young, chronic fatigue syndrome and various dermatological conditions [2].

One such rare disease associated with COVID infection is the occurrence of mucormycosis.

Case reports of sporadic RSCM have been reported in the past with the earliest reports by Kurrien [3] in 1954 and Smith et al. [4] in 1958.

Rhinosinocerebral mucormycosis (RSCM) is a rare infection, caused by several fungal species such as *Mucor, Cunninghamella, Rhizopus* and *Lictheimia,* usually associated with immunocompromised patients and diabetic individuals [5]. One of the largest outbreaks of RSCM occurred in India close to the second wave of the SARS-CoV-2 infection [6].

RSCM presents with ophthalmic and non-ophthalmic manifestations.

Non-ophthalmic manifestations of RSCM include sinusitis (100%), nasal discharge/ulceration (74%), infranuclear abducens nerve palsy (46%), palatal necrosis (29%), cerebral lobe involvement (20%), and hemiparesis (17%). Orbital involvement was observed in 80% of patients with cavernous sinus thrombosis in 11%, and internal carotid occlusion (ICA) and hydrocephalus in 3% each [7].

Involvement of cranial nerves by mucormycosis, signifying intracranial extension, generally carries poor prognosis [8, 9]. Imaging however allows early identification of cranial nerve involvement and exact delineation of the spread of infection. This allows aggressive medical and surgical management of these complications leading to an overall improvement in the outcome.

## Methods

Inclusion criteria for the subjects included subjects consenting for the study, subjects with two or more cranial nerve involvement and those with history of COVID-19 infection preceding mucor infection.

Subjects excluded from the study included those with just a single cranial nerve involvement, pregnant patients, those with mucor infection not preceded by COVID-19 infection and patients less than 18 years of age.

**Fig. 1:**
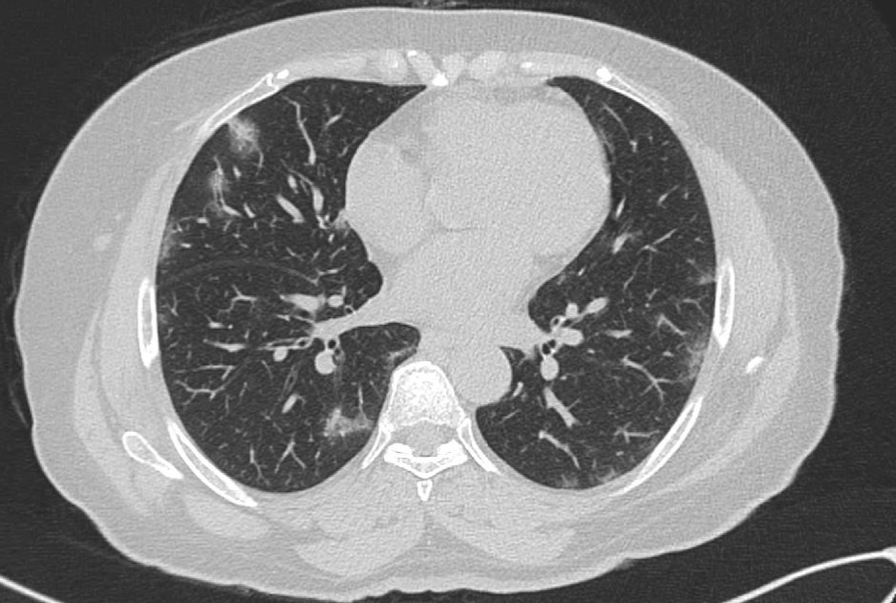
Axial HRCT thorax image showing ground-glass opacities in subpleural and peripheral regions of bilateral lungs typical for COVID pneumonitis

Seven patients of invasive fungal disease with multiple cranial nerve involvement were studied. All seven patients in our study had a prior history of COVID-19 infection. Nasal swabs, in all seven cases, were positive for COVID-19 infection by polymerase chain reaction. Reverse transcriptase-polymerase chain reaction (RT-PCR) from nasopharyngeal swabs for COVID-19 was positive in all subjects. Further, computed tomography (CT) scan of the thorax showed changes typical for COVID-19 pneumonitis in all cases. None of the subjects had received any doses of the COVID vaccine at the time of the disease (Fig. 1).

**Fig. 2:**
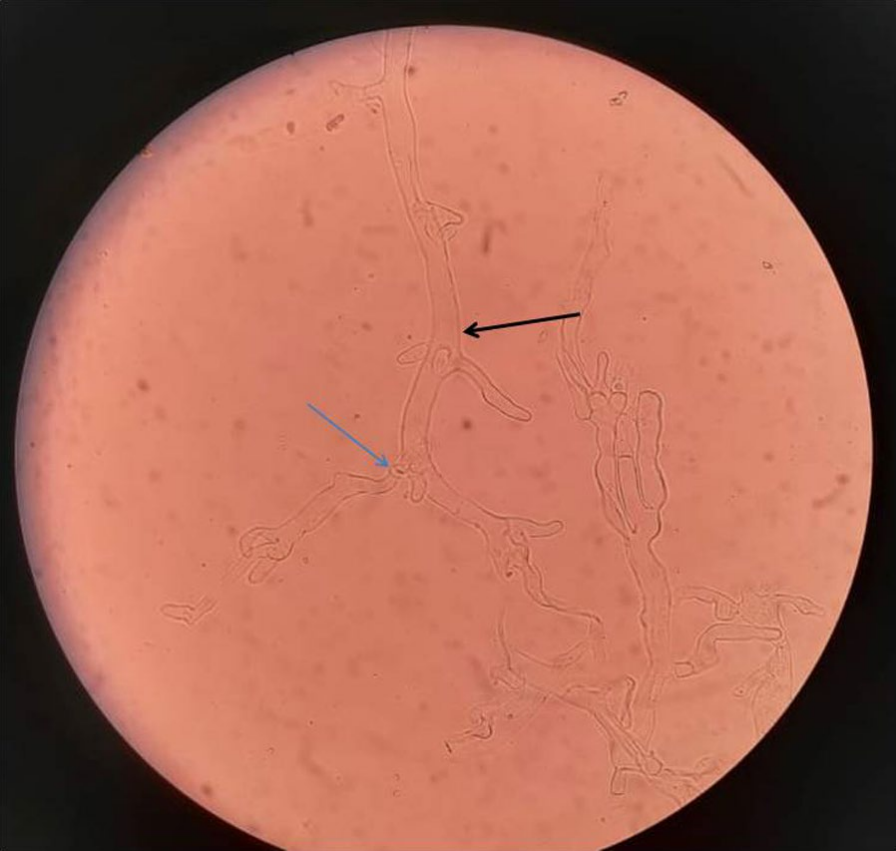
Potassium hydroxide (KOH) mount showing ribbon-shaped aseptate hyphae (black arrow) showing branching at right angle (blue arrow)

**Fig. 3:**
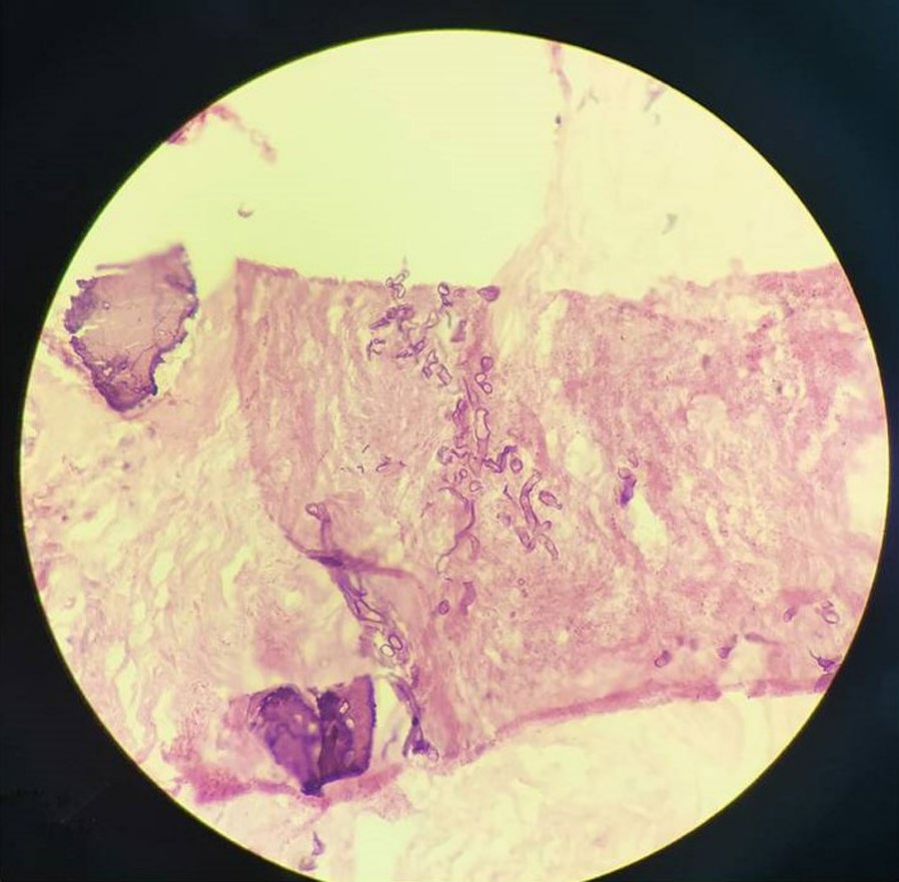
Hematoxylin and eosin stained microsections showing fragments from the sinonasal mucosa with broad aseptate ribbon-like fungal hyphae showing branching at right angles consistent with *Mucor* species

Evaluation at presentation included a detailed history, otorhinological, ophthalmology, and neurology examination to assess the extent of disease, sinonasal endoscopy with biopsy. Diagnosis was made on KOH mount (Fig. 2) and histopathological examination (Fig. 3).

Magnetic resonance imaging (MRI) was performed using 3 Tesla (T) Siemens (Verio: Siemens Medical System, Erlangen, Germany). MRI of paranasal sinuses (PNS) and brain were obtained to assess the extent of disease. Contrast-enhanced MRI (CE-MRI) protocol of the PNS, brain, and orbits included T2 FLAIR, GRE, DWI, axial T1, T2, STIR coronal, CISS, T1 FS axial post-contrast (3 mm thickness) and T1 FS post-contrast coronal (3 mm thickness) sequences. Patients were started on systemic amphotericin B at the diagnosis of mucormycosis. All the patients underwent surgical debridement for the necrosed tissue. Of these two patients underwent functional endoscopic sinus surgery (FESS) and enucleation of the left orbit and two patients underwent FESS before the MRI was done.

The MRI scans of the subjects were interpreted and reviewed by two radiologists independently, specializing in head, neck and face radiology and neuroradiology and each with an experience of about 5 years.

## Case information

**Fig. 4:**
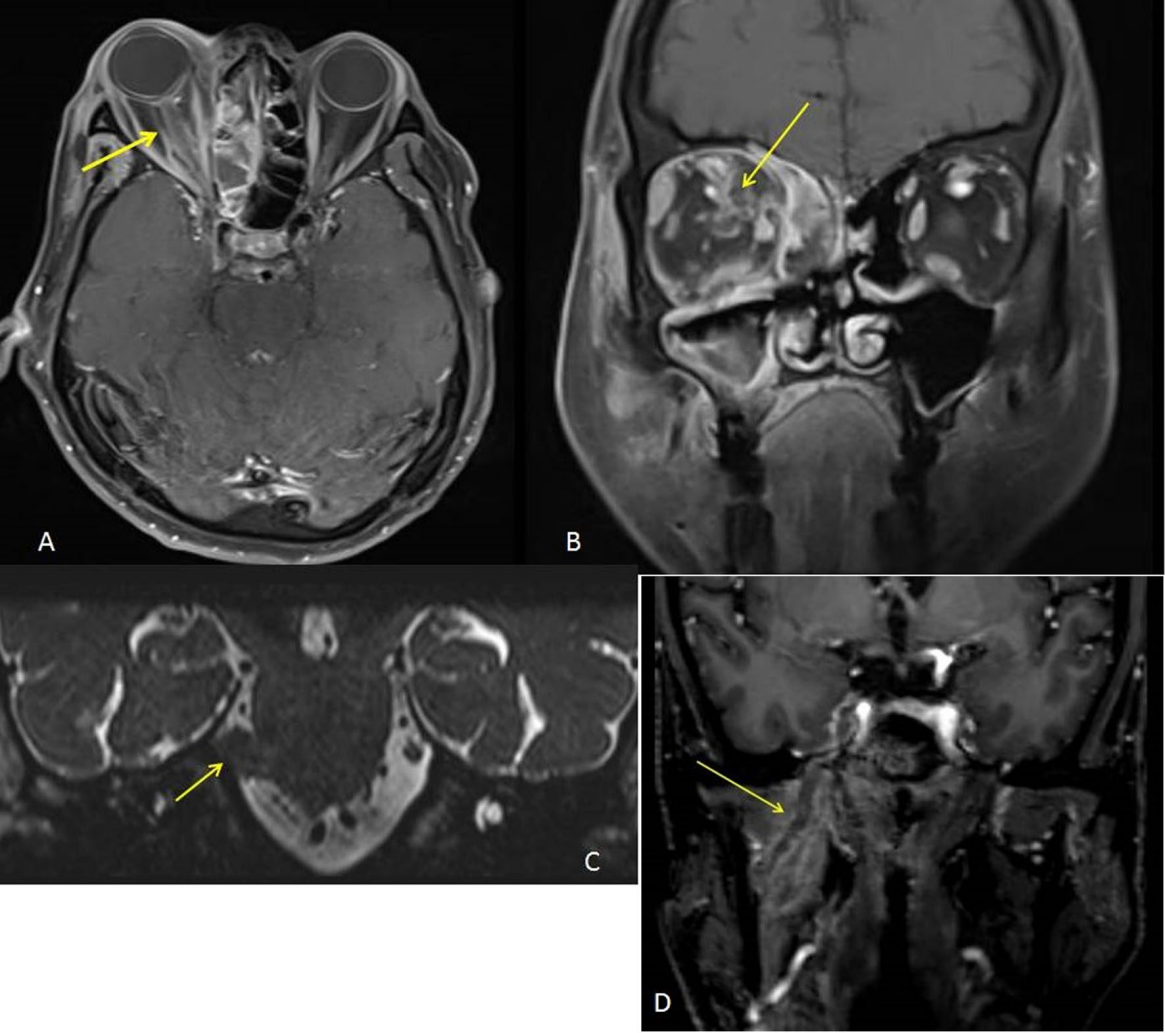
**A**, **B** T1 post-contrast sequence in axial and coronal planes showing optic neuritis with changes of orbital cellulitis on the right side. **C** CISS in coronal plane shows thickened right trigeminal nerve. **D** T1 post-contrast sequence in coronal plane showing extensive post-contrast enhancement in the pterygoid muscles along the inferior alveolar nerve on the right side

*Case 1* A 32-year-old man, presented with right-sided facial swelling, lower toothache, painful proptosis of the right globe and loss of vision. MRI showed optic neuritis with trigeminal nerve abscess and extension of inflammation along the inferior alveolar nerve (Fig. 4).

**Fig. 5:**
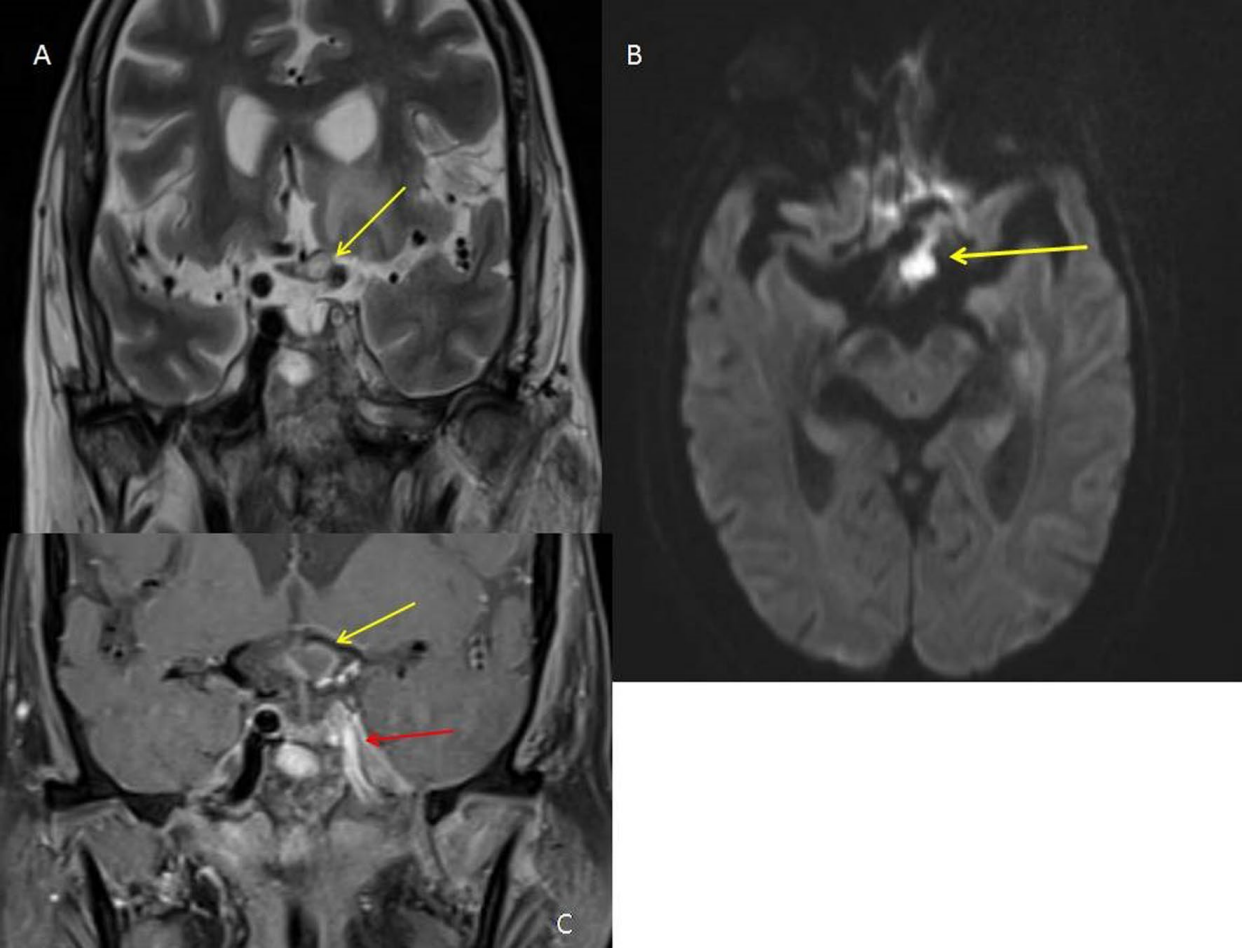
**A** STIR coronal sequence demonstrating hyperintense residual disease (arrow) in the left half of optic chiasma. **B** Axial diffusion-weighted sequence demonstrating diffusion restriction in the left half of optic chiasma. **C** T1 post-contrast coronal sequence showing peripheral contrast enhancement (yellow arrow) in the left optic nerve. Also noted is the loss of flow void (red arrow) of the cavernous portion of the left ICA, suggestive of thrombosis

*Case 2* A 73-year-old lady, presented with left-sided facial swelling, and left orbital cellulitis, due to mucormycosis. The patient subsequently underwent enucleation and in the postoperative period had purulent discharge from the orbit. MRI confirmed the presence of disease in the optic chiasma along with thrombosis of the cavernous portion of the left ICA (Fig. 5).

**Fig. 6:**
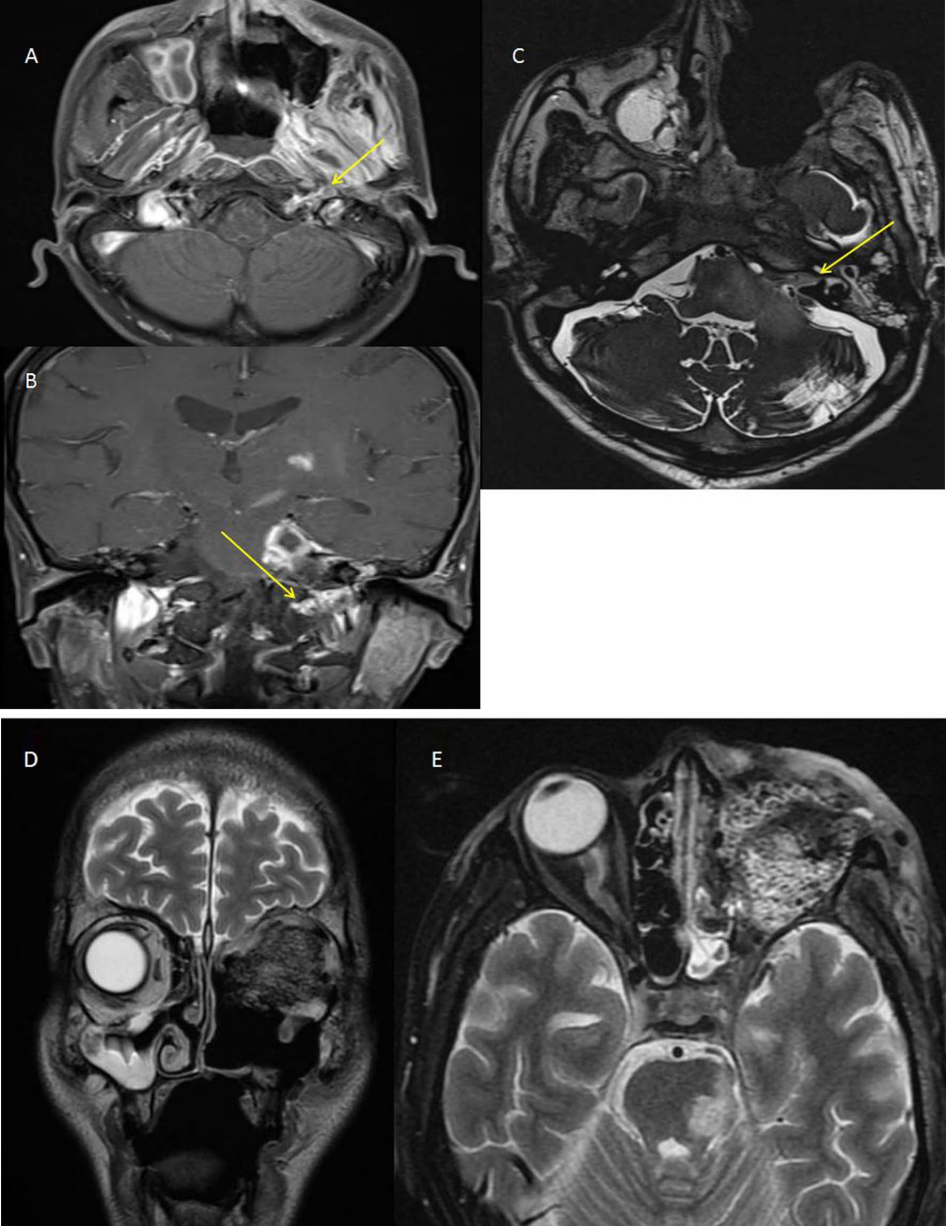
**A** T1 post-contrast axial sequence and **B** post-contrast coronal sequence showing post-contrast enhancement along the left hypoglossal nerve (arrows). MRI showing inflammation along the left CP angle with involvement of seventh and eighth cranial nerves as a result. Thickening and extension of inflammation were seen along the internal acoustic meatus, hypoglossal canal and the left inferior alveolar canal with VII, VIII and XII involvement. **C** CISS in the axial plane demonstrating thickening of the cisternal segments of the seventh-eighth cranial nerve complex on the left side. **D** T2 axial and **E** coronal sequences showing hypointense fungal tissue in the left orbit

*Case 3* A 34-year-old male, presented with sudden onset left-sided facial palsy with painful proptosis of the left eye and complete ophthalmoplegia. A thorough clinical examination revealed that the patient had a hearing deficit in the left ear (which subsequently proved to be of sensorineural type). On asking the patient to protrude his tongue for examination, the tongue was found to deviate toward the left side (involved side), implying left-sided hypoglossal nerve palsy. No evidence of motor or sensory disturbances were found. A clinical diagnosis of Garcin syndrome secondary to fungal infection was made (Fig. 6).

**Fig. 7:**
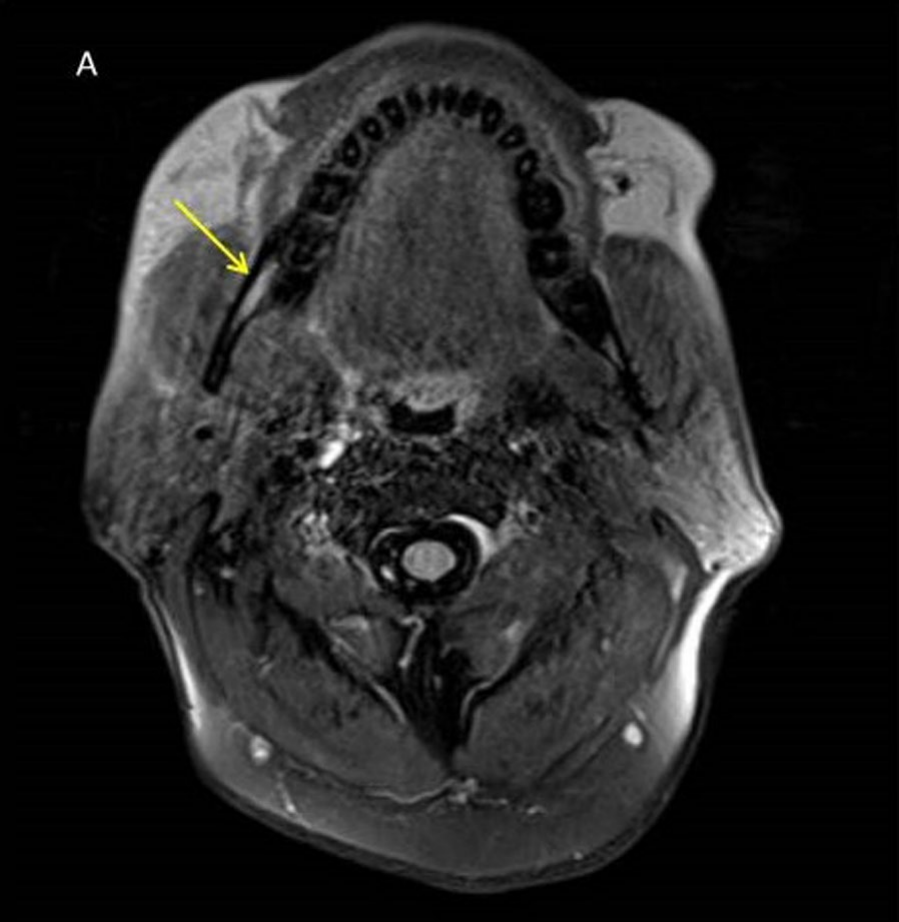
T1 post-contrast axial sequence showing thickened enhancing right inferior alveolar nerve (arrow)

*Case 4* A 60-year-old male presented with headache, painful proptosis and complete ophthalmoplegia of the right eye. MRI brain was suggestive of right orbital cellulitis, with the inflammation seen extending posteriorly till the orbital apex with optic peri-neuritis. The right inferior alveolar nerve was found to be thickened and showing abnormal enhancement (Fig. 7).

**Fig. 8:**
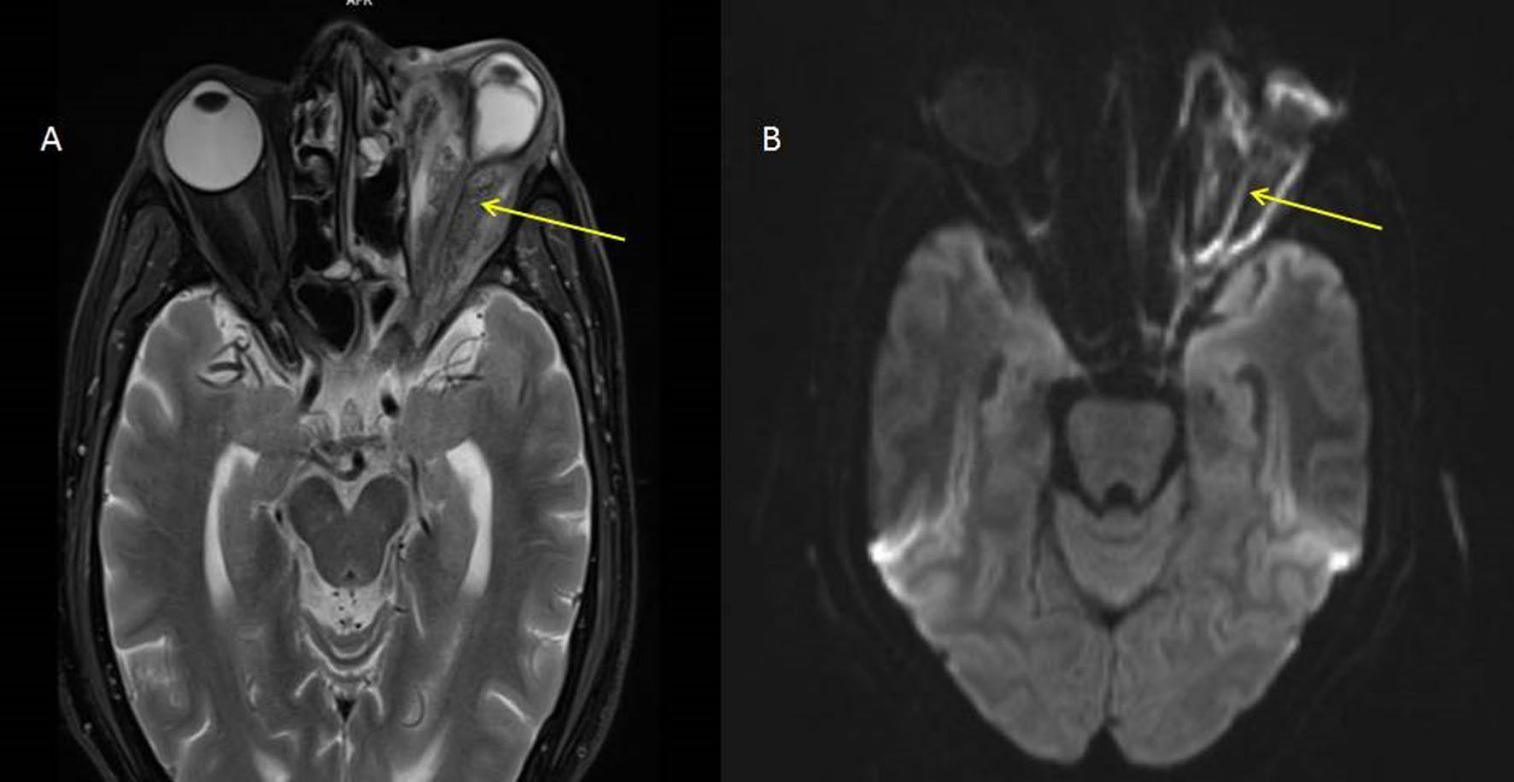
**A** T2 axial sequence showing changes of orbital cellulitis on the left side with abnormal signal intensity in the intraorbital portion of the left optic nerve. Also noted is the T2 hypointense fungal tissue infiltrating the left orbit and causing severe distortion of the left globe. **B** Axial diffusion sequence showing diffusion restriction along the intraorbital portion of the left optic nerve

*Case 5* A 62-year-old male, presented with a left eye proptosis and acute loss of vision. Extensive left orbital cellulitis with diffusion restriction seen within the optic nerve suggests acute ischemic optic neuropathy. Infiltration was noted along the mandibular division trigeminal nerve (V3) through the left foramen ovale and the Meckel’s cave (Fig. 8).

*Case 6* A 59-year-old man, presented with left eye proptosis and acute loss of vision. MRI showed extensive left orbital cellulitis, with left optic neuritis with inflammation seen reaching up to the orbital apex. Thickening of the seventh-eighth nerve complex was also seen on the left side.

**Fig. 9:**
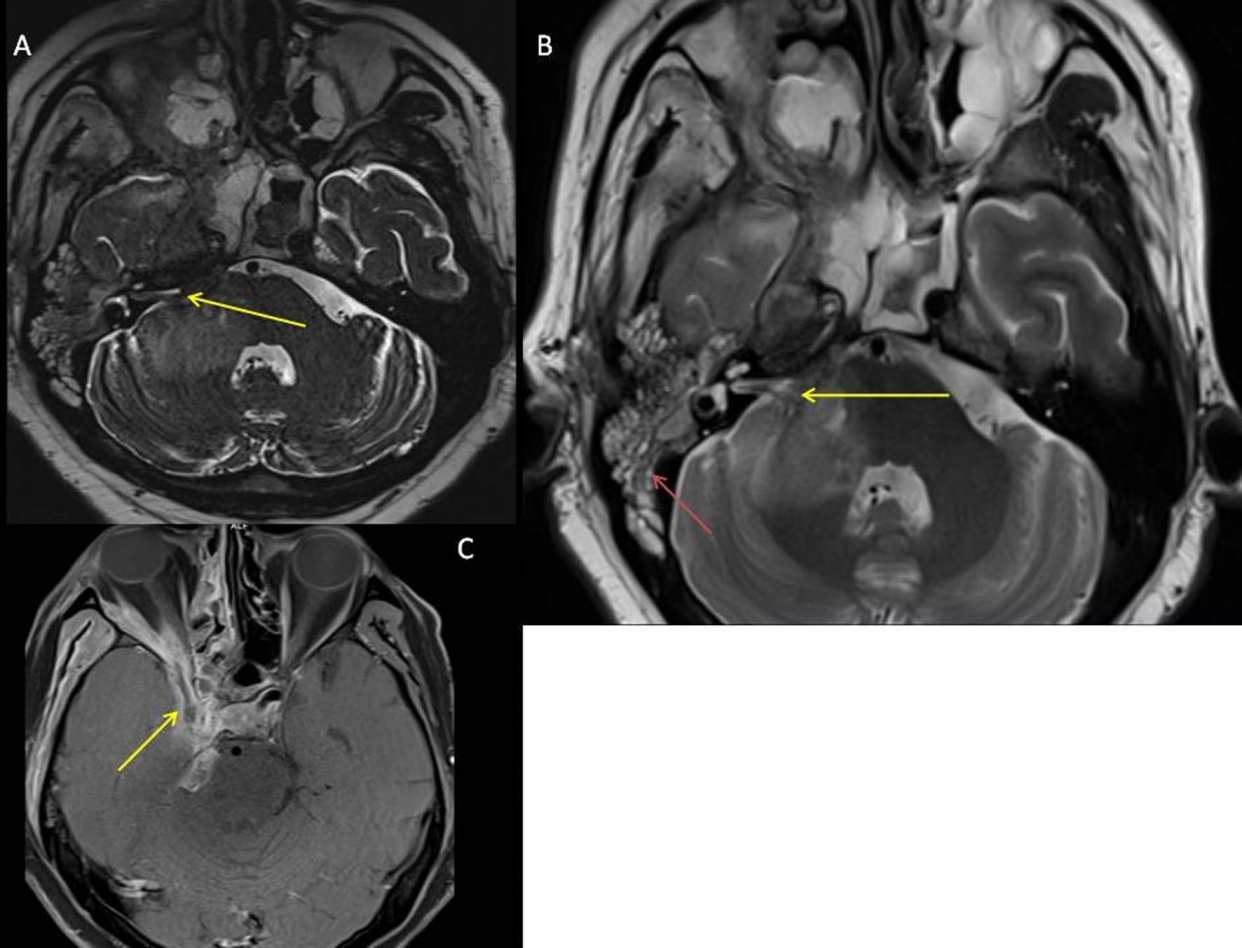
**A** CISS, and **B** T2 WI sequence showing thickening of the cisternal segment of the seventh-eighth nerve complex on the left side. The underlying right middle cerebellar peduncle shows T2 hyperintense signal. Fluid signal is noted replacing the normal mastoid air cells on the right suggestive of mastoiditis (red arrow). Inflammation is noted along the floor of the left orbit and sphenoid sinusitis. **C** T1 WI post-contrast axial sequence showing abnormal enhancement along the right cavernous sinus extending into the orbit

*Case 7* A 38-year-old man, presented with proptosis of the right globe, with redness and chemosis. MRI brain showed orbital cellulitis with optic neuritis on the right side. Involvement of cavernous sinus thrombosis with involvement of trigeminal nerve and subtle thickening of the seventh-eighth nerve complex on the right side. An ill-defined abscess was seen in the right temporal lobe and the right half of the pons (Fig. 9).

## Results

The results and observations gathered can be summarized in Table 1.

**Table 1:** Master chart for the cases

	Case 1	Case 2	Case 3	Case 4	Case 5	Case 6	Case 7
Age	32-years	73-years	34-years	60-years	62 years	59 years	38 years
Gender	Male	Female	Male	Male	Male	Male	Male
Time interval between COVID and mucormycosis	4 weeks	10 weeks	1 week	1 week	3 weeks	2 weeks	3 weeks
History of steroid use	+	+	+	+	−	−	+
Presence of diabetes mellitus	−	−	−	−	+	+	−
Clinical features on presentation	Facial swelling, proptosis, and loss of vision	Facial swelling, reduced visual acuity	Facial palsy, ophthalmoplegia	Headache, proptosis, ophthalmoplegia	Ocular proptosis and loss of vision	Ocular proptosis and loss of vision	Ocular proptosis and facial swelling
Histopathology and KOH mount	Aseptate hyphae	Aseptate hyphae	Aseptate hyphae	Aseptate hyphae	Aseptate hyphae	Aseptate hyphae	Aseptate hyphae
Optic nerve	+	+	+	+	+	+	+
Oculomotor nerve			+				
Trochlear nerve			+				
Trigeminal nerve	+	+	+		+		+
Facial nerve			+			+	+
Vestibulocochlear nerve			+			+	+
Hypoglossal Nerve			+				
Inferior alveolar nerve	+		+	+			
Internal carotid artery thrombosis		+	+				+

+ means present, − means absent

Out of the seven subjects who were included in the study, six patients were males, while one was female. The ages of the participants spanned from 32 to 73 years. All, but two had a history of steroid use while they were undergoing treatment for COVID infection. The other two patients had a history of diabetes mellitus (> 5 years each), which was largely under control. The glycosylated hemoglobin (HbA1c) of these patients was < 7%.

The mean time from the diagnosis of COVID infection to the occurrence of mucormycosis in the patients was found to be 3.7 weeks. The optic nerve was the most frequently affected. This was followed by the involvement of trigeminal nerves and its branches. Thrombosis of the intracranial portion of the ICA was seen in three patients. The most common imaging feature of fungal neuritis was abnormal thickening and enhancement of the cranial nerve with perineural fat stranding. All cranial nerves in a particular subject were found to be involved only on one side (unilateral affection) and there were no cases wherein bilateral affection by the disease was found.

## Discussion

The coronavirus disease 19 (COVID-19) is a highly transmittable and pathogenic viral infection with multiple variants [10, 11] caused by severe acute respiratory syndrome coronavirus 2 (SARS-CoV-2), which caused global pandemic leading to healthcare crises and severe loss of human life. Fungal infections, including mucormycosis, aspergillosis and invasive candidiasis, have also been reported in patients with severe COVID-19 or those recovering from the disease. India had seen a rise in the rhino-orbital mucormycosis co-infections in COVID-19 patients during the second wave of COVID-19 infection [6]. Mucorales are killed by the mononuclear and polymorphonuclear phagocytes of normal hosts by generation of oxidative metabolites and defensins. This makes patients with neutropenia, those with dysfunctional phagocytes and uncontrolled diabetes particularly susceptible toward the development of invasive mucormycosis [12]. COVID-19 causes profound lymphopenia and in advanced infections viral replication accentuates the inflammatory response and neutrophil and monocyte influx in the bloodstream [13]. This in turn leads to an imbalance between neutrophil and lymphocyte action making the patient more susceptible to systemic fungal infections [14]. Further, in a study conducted by Wu et al. [15], it has been postulated that SARS-CoV-2 infection induces apoptosis of β cell of the pancreatic islets thereby attenuating insulin levels and secretion thereby making COVID-19 infection a potential diabetogenic state [16].

Further, it has been found in several studies that administration of steroids, and biologics such as tocilizumab have been known to lead to fungal infections and candidemia [17, 18].

Mucormycosis tends to manifest in one or more of these six forms—Rhinosinocerebral, pulmonary, cutaneous, gastrointestinal and miscellaneous with RSCM being predominant in patients with uncontrolled diabetes and the pulmonary form being noted in patients with hematological malignancies and solid organ transplant recipients [5].

RSCM typically starts with nasal involvement and spreads to adjacent sinuses [19, 20]. Around the paranasal air sinuses, there are many skull base foramina and fissures through which these cranial nerves traverse. From the infected paranasal sinuses, the fungal elements can take these avenues to enter the skull base and intracranial compartment. Whilst doing so, within these channels, the fungi affect, infect, invade, infiltrate and may destroy these important structures and result in various cranial nerve palsies. Anosmia, visual loss, ocular palsies, trigeminal neuralgia, and even lower cranial palsies can occur [21–24].

High fatality is because of angioinvasion by mucormycosis with resultant thrombosis of vessels and resultant tissue necrosis [25]. Three patients in the above series also developed cerebral infarcts secondary to ICA thrombosis.

MRI is an indispensable tool that can be used to diagnose mucormycosis infections involving sino-nasal region, orbits, and intracranial extension of the disease. MRI is useful in assessing the intradural extent of the disease, perineural spread, and thrombosis of the cavernous portion of the ICA, caused by fungal infection as well as planning of the surgical margins for resection and post-surgical follow-up [26, 27].

Sino-nasal mucormycosis on MRI is seen as mucosal thickening, which appears hypointense on T1WI and variable to hyperintense on T2WI [27]. Fungal tissue appears hypointense on T2WI and may show restricted diffusion on DWI. The involved tissues show post-contrast enhancement; however, areas of non-contrast enhancement are noted within the enhancing turbinates and paranasal sinuses known as the black turbinate sign [28].

MRI is the most sensitive modality to look for cranial nerve involvement due to its superior soft tissue resolution. MRI findings of cranial nerve involvement in mucormycosis may be as subtle as perineural fat stranding in the early stages, or may show thickening and enhancement of the cranial nerves. There may be meningeal enhancement or phlegmonous soft tissue noted along the cranial nerves. Changes of denervation may be seen in the muscles innervated by the cranial nerve, which include T2W hyperintensity and enhancement in the early stages and fatty changes with volume loss in long-standing denervation [29]. In some cases, when the nerve involvement is subtle, these denervation changes can provide a clue to neural involvement.

## Limitations of the study

This series being a pilot study covers very few patients. Secondly, fungal infection affecting all cranial nerves could not be demonstrated in this study. In case 3, MRI imaging could not convincingly demonstrate affection of the lower cranial nerves, namely, spinal accessory, vagus and the glossopharyngeal nerves.

## Conclusion

Mucormycosis is a serious disease and may be fatal if not treated in time. A high index of clinical suspicion, early diagnosis, confirmation with microbiology, staging with cross-sectional imaging and immediate correction of underlying medical disorders and institution of antifungals are important factors for a favorable prognosis. We have highlighted cranial nerve involvement in post-COVID mucormycosis in this case series. Identifying cranial nerve involvement is important in diagnosis and staging of mucormycosis. MRI is the modality of choice for early identification and precise delineation of the extent of disease.

## Data Availability

The data and materials supporting the findings of this study are available on request from the corresponding author.

## Abbreviations

3 T: 3 Tesla
CISS sequence: Constructive interference in steady-state sequence
COVID-19: Coronavirus disease 2019
CT: Computed tomography
DWI: Diffusion-weighted imaging
FESS: Functional endoscopic sinus surgery
FLAIR: Fluid-attenuated inversion recovery
FS: Fat saturated
GRE: Gradient recalled echo
HbA1C: Glycosylated hemoglobin
ICA: Internal cerebral artery
KOH: Potassium hydroxide
MRI: Magnetic resonance imaging
PNS: Paranasal sinuses
RSCM: Rhinosinocerebral mucormycosis
RT-PCR: Reverse transcriptase polymerase chain reactions
SARS-CoV-2: Severe acute respiratory syndrome coronavirus 2
STIR: Short tau inversion recovery sequence
T1WI: T1 weighted image
T2 WI: T2 weighted image
V3: Mandibular branch of trigeminal nerve
